# The Effect of Educational Intervention on the Improvement of Nontechnical Skills in Circulating Nurses

**DOI:** 10.1155/2021/5856730

**Published:** 2021-10-15

**Authors:** Reza Kalantari, Zahra Zamanian, Mehdi Hasanshahi, Seyed Aliakbar Faghihi, Jamshid Jamali, Hadi Niakan

**Affiliations:** ^1^Department of Ergonomics, Faculty of Public Health, Shiraz University of Medical Sciences, Shiraz, Iran; ^2^Department of Operating Room, School of Nursing, Shiraz University of Medical Sciences, Shiraz, Iran; ^3^Clinical Educational Research Center, Shiraz University of Medical Sciences, Shiraz, Iran; ^4^Department of Biostatistics, School of Health, Mashhad University of Medical Sciences, Mashhad, Iran; ^5^Department of Surgery, Faculty of Medicine, Shiraz University of Medical Sciences, Shiraz, Fars, Iran

## Abstract

**Background:**

Nontechnical skills are necessary for clinicians' safe performance and prevention of errors in the operating room. Educational intervention is a useful way to improve these skills, which are a vital area for improvement. Circulating nurses are surgical team members whose work depends heavily on using nontechnical skills. This study is aimed at assessing the effect of an educational intervention on the improvement of circulating nurses' nontechnical skills.

**Methods:**

This semiexperimental study was conducted on 300 circulating nurses divided into the intervention and no intervention groups each containing 150 participants. The nontechnical skills were assessed using the circulating practitioners' list of nontechnical skills. Then, the intervention group received training regarding these skills, and the two groups were evaluated again. After all, the data were entered into the SPSS 24 software and were analyzed using descriptive statistics and Wilcoxon and Mann–Whitney tests. Furthermore, Kendall's tau, independent sample *t*-test, and one-way ANOVA were used for assessment of relationship between median scores and demographics.

**Results:**

The results revealed a significant improvement in the scores of all domains of nontechnical skills in the intervention group (*p* < 0.05). The highest and lowest improvements were observed in teamwork (42%) and situational awareness (16.7%), respectively. After the intervention, the scores of some of the behaviors were still below the average level or were not improved significantly.

**Conclusions:**

Circulating nurses' nontechnical skills can be improved by educational interventions. However, regarding the low scores or no improvements in the scores of some behaviors, other intervention types such as policymaking and correcting the existing hierarchies in the operating room can be useful to complete the educational interventions.

## 1. Introduction

Nontechnical skills refer to the intrapersonal and cognitive skills that facilitate the effective delivery of safe services [1]. Healthcare staff often work in high-demand work settings [2]. Adverse events (unexpected medical problems that happen during treatment) in surgeries have highlighted the importance of nontechnical skills in the operating room [3]. Surgical procedures are known as standard treatment for many illnesses [4–7], so studying nontechnical skills in surgeries is of interest. Deficiencies in these skills have been found to be associated with a higher risk of surgical complications and errors [8]. In a study, half of the fatal medical accidents happened due to poor nontechnical skills [9]. Approximately 86% of adverse events in open surgeries also resulted from deficiencies in nontechnical skills [10]. Adequate nontechnical skills can decrease the chance of errors and increase patient safety [11]. These skills can improve technical skills in stressful situations [12] and are essential to an effective surgery [13]. Communication, teamwork, situational awareness, leadership, and task management are some of the categories of nontechnical skills [14].

Nontechnical skills are a part of formal training in some high-risk work settings such as aviation industries and operating rooms [15]. Formerly, the surgical curricula were directed towards the acquisition of technical skills [16]. Nowadays, nontechnical skills are included in surgical curricula [17], as these skills have emerged as a vital area for improvement in this domain [18]. However, numerous studies on nontechnical skills in the operating room have reported suboptimal or poor skill levels [8]. Therefore, there is a need for improvement in this domain. In fact, safe healthcare practice depends on nontechnical skills. Consequently, healthcare educators have focused on training programs regarding these skills [1]. These skills have been regarded as catalysts for improving personal competence [19] and technical performance [20]. Many quality improvement programs have been designed to improve nontechnical skills, and most of them have reported positive outcomes [1]. Cognitive training, classroom teaching, simulation, and ward-based scenarios are the most popular training methods that have already been used for improving nontechnical skills [18].

Circulating nurses are surgical team members who have important duties in the operating room [14]. Conducting safety checks in the operating room, monitoring the sterile area, and positioning patients are among circulating nurses' duties [21], which depend on the utilization of nontechnical skills [22]. Very little is currently known about circulating nurses' nontechnical skills [23]. Prior studies have revealed different findings in terms of circulating nurses' nontechnical skills. It has been indicated that they can do well enough to recover errors during surgical processes [24]. They can also keep calm under stressful situations and solve problems in the operating room [25]. However, it was demonstrated that circulating nurses had the most concerning communicational patterns in the operating room [26]. The results of another study showed their role in communication breakdowns, which led to the retention of surgical instruments within the patient's body [27].

During the recent decades, training nontechnical skills has received considerable attention [28]. Former studies on training nontechnical skills among the operating room staff were conducted on the whole surgical team [29, 30] or surgical team members [31, 32]. However, no study has been conducted on the improvement of nontechnical skills among circulating nurses. Considering the special role of circulating nurses in the operating room, it seems necessary to assess and promote their nontechnical skills, as nontechnical skill training can be helpful in resolving failures and errors [33]. Previous studies recommended educational interventions in this regard. These studies tried to improve the nontechnical skills of surgical team members using seminars, classroom courses, face-to-face education, etc. [34, 35]. The present study is aimed at assessing the effect of an educational intervention on the improvement of circulating nurses' nontechnical skills.

## 2. Methods

This research was approved by the Ethics Committee of Shiraz University of Medical Sciences. This semiexperimental study was conducted on the circulating nurses who worked in the operating rooms of four main public hospitals affiliated with Shiraz University of Medical Sciences. All the circulating nurses who worked in the mentioned hospitals were invited to participate in the study. Generally, the circulating nurses who work in Iranian hospitals are operating room technicians. They study for at least two years before working in operating rooms. They should be able to work as a scrub nurse, too.

**Figure 1:**
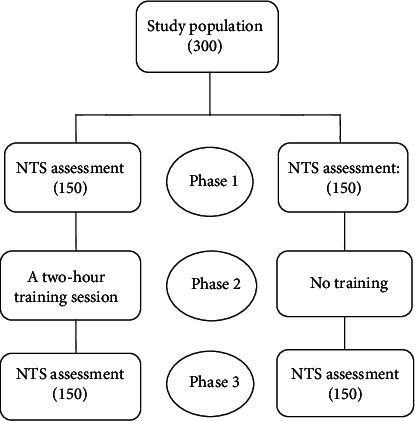
The study design flowchart.

The inclusion criteria of the study were being willing to participate in the study and having no experience of being trained regarding nontechnical skills in an educational program. The trainees were excluded from the study since their presence could decrease the quality of the training for seniors [36]. Out of the 316 circulating nurses, 300 were eligible to participate in the study. The participants were divided into two groups (intervention and no intervention) based on the random selection of work shifts during a month in order to prevent heterogeneity between the groups that were blinded to the study design. The study design has been depicted in Figure 1.

The data were collected using a demographic questionnaire including questions about age, work experience, gender, and education level and the circulating practitioner's list of nontechnical skills (CPLINTS) observational tool. This valid and reliable tool contained 43 observable behaviors grouped into five dimensions, namely, task management, teamwork, situational awareness, communication, and leadership. Each domain was composed of several elements. The elements of the domains have been presented in Table 1.

Each item could be scored from zero (necessary but not observed) to four (excellent). There was also a “not applicable” option for some of the behaviors. The mean scores of the behaviors could be calculated for each dimension of nontechnical skills [14]. The description of rating options for each behavior has been presented in Table 2.

Before the beginning of data gathering, the authors gained permissions from the hospitals' managers. They entered the operating room before the beginning of surgery, introduced themselves, and described the aims of the study to the participants. Written informed consent was also obtained from the participants. In the first phase of the research, the circulating nurses were observed during the surgery process. The circulating nurses were rated by an observer with seven years of experience in studying nontechnical skills in the operating room. It should be noted that the observer remained silent without disrupting the surgical team's activities during data collection.

An educational intervention was designed by the research team, and a face-to-face approach was used. A human factors specialist with eight years' experience in studying and educating nontechnical skills conducted this step. In the intervention group, the circulating nurses were trained regarding the definition of nontechnical skills, importance of these skills in preventing surgical errors, and CPLINTS domains and behaviors. The circulating nurses were also trained in terms of task management, communication, teamwork, leadership, and situational awareness domains. Furthermore, each participant's data from the first phase of the study were used, so that the intervention provided feedback regarding the observed behaviors in the first phase. Real case discussion has been found to be useful in nontechnical skill training [37]. The training sessions were held in the operating rooms during the circulating nurses' rest times. Each session lasted for two hours. Eight weeks after the intervention, the first phase was repeated and the circulating nurses were rated.

The SPSS 22 software was used for data analysis. The results of the Kolmogorov-Smirnov test showed that the distribution of the scores of nontechnical skills was not normal. Thus, nonparametric statistical tests were used in this study. Median and interquartile range were used for describing the scores. In addition, Wilcoxon and Mann–Whitney *U* tests were used for assessing the effectiveness of the intervention in nontechnical skills. Kendall's tau coefficient was used for assessment of correlations between differences in pre-post scores and age and work experience years. The independent sample *t*-test was used for comparing the median of difference scores between men and women nurses. Furthermore, the one-way ANOVA test was used for comparing the differences of median scores among circulating nurses with different university education levels. The significance level was set at 0.05.

## 3. Results

The demographic information of the study population has been presented in Table 3.

The median scores of nontechnical skills were 2.26 and 2.73 (out of 4) in the no intervention and intervention groups, respectively, after implementing the educational program. The overall scores of nontechnical skills were significantly improved after the educational intervention (*p* < 0.001). The median scores of the domains of nontechnical skills and their differences within each group have been presented in Table 4. The Mann–Whitney *U* test results have been included for comparing after intervention scores between two groups, as well.

The percentages of improvement in the domains' scores in the intervention group were calculated by dividing the postintervention scores to preintervention scores in the intervention group. The results have been shown in Table 5.

The results of the Wilcoxon test demonstrated a significant improvement in the scores of all the elements of each nontechnical skill domain in the intervention group (*p* < 0.01). All elements and items of situational awareness were improved significantly after the intervention. Management of communication, assertiveness, preparing and planning the stage, coping with pressure, and cooperation were the elements of nontechnical skills that were improved significantly after the intervention. The significantly unimproved behaviors after the educational intervention or the behaviors that still had lower scores than average belonged to only four elements, namely, exchange of information, operating room management, maintaining standards, and cooperation. These behaviors included helping the anesthesia team, controlling the traffic of clinicians in the operating room, adequate information transfer during hand-offs, solving problems in the operating room, reacting properly to annoying noises, staying in the operating room until the end of the surgery, asking questions to gain information, and not using mobile phones in the operating room. Avoiding unnecessary escapes from the operating room and not using mobile phones during surgery were the behaviors with the lowest scores.

The relationship between the demographics and the differences in the median scores of nontechnical skills before and after the intervention in the intervention group was studied. The results revealed the role of demographics in the effectiveness of the intervention. Age and work experience had a significantly strong negative relationship with the differences in the overall scores. Kendall's tau coefficient and *p* value for age were -0.654 and <0.001, respectively. These measures were, respectively, obtained as -0.658 and <0.001 for work experience. Moreover, females had significantly better difference median scores than men (0.64 against 0.46) after the intervention (*p* = 0.004). Furthermore, the nontechnical skill difference median scores were significantly better (*p* < 0.001) among the circulating nurses with Bachelor's degrees (0.64) compared to those with Associate degrees (0.26).

## 4. Discussion

This pre-post study is aimed at assessing the effect of an educational intervention on the circulating nurses' nontechnical skills. The results indicated that the educational intervention increased the scores of the nontechnical skills significantly. However, some of the behaviors were not significantly improved after the intervention or still had lower-than-average scores.

Nursing professions are important areas for research [38]. To the best of our knowledge, this was the first interventional study on the improvement of circulating nurses' nontechnical skills at the individual level by training. In this study, training the circulating nurses in terms of nontechnical skills increased their total scores of nontechnical skills significantly, which was in line with the results of the study performed by McCulloch et al. [29]. Using a structured framework to train the circulating nurses, real case discussions, and giving necessary feedbacks regarding their behaviors were helpful in improvement of these skills. Considering the increase in the scores of different nontechnical skill domains using educational interventions, similar results have been obtained in most previous studies [1]. For instance, training nontechnical skills could improve the safety culture in clinicians by shifting the norms of acceptable behaviors [39]. Applying crew resource management-based training programs was also a helpful way to improve nontechnical skills [40].

The current study findings indicated that all items of situational awareness were improved significantly after the intervention. This could be attributed to the usefulness of training regarding other nontechnical skills such as task management [41, 42] in the improvement of situational awareness skills. This result was in agreement with that of the study conducted by McCulloch et al., in which situational awareness was significantly improved among operating room nurses [29]. Training situational awareness is necessary to improve safety in the operating room [43]. Additionally, properly checking the operating room elements is crucial for preventing patient- and instrument-related problems. Improvements in paying attention to the surgical process and anticipating the near future can be helpful in the smooth flow of surgeries and prevention of delays.

Operating room nurses should be able to work within the surgical team [35]. In the present study, the educational intervention improved the circulating nurses' cooperation and coordination abilities with other team members. A similar result was obtained in the research performed by Bleakley et al. on the effect of an educational intervention on teamwork scores [44]. It is very important for circulating nurses to coordinate with other team members, for example, in counting tasks. A previous study revealed that circulating nurses were really concerned about teamwork [45]. In that study, intervention was reported as a useful way to improve these nurses' skills. The only item in this domain that was not improved after the intervention was cooperation with the anesthesia team. This could result from these two groups' different workstations or different hospital routines affecting the relationships among the practitioners of different subteams.

The present study findings showed improved communication among the circulating nurses after the intervention, which was consistent with the results obtained by Takala et al. [46]. Accordingly, the circulating nurses did very well in all the items of communication management after the training. This issue is of paramount importance, because sometimes speaking quietly, speaking on time, and even remaining silent are vital and can be more helpful than communication [47]. The two items that were not improved were asking and giving necessary information in different situations, while hand-off communications are vital for ensuring patient safety and preventing surgical complications [48]. The use of other intervention methods such as simulation-based training is a helpful way to improve hand-off communication [49].

The leadership domain in circulating nurses consisted of two elements. In the current investigation, the circulating nurses' assertiveness was improved significantly after the intervention, while managing the operating room situation was not improved. Since distributed leadership in the operating room can be helpful [50], circulating nurses should show limited leadership. Assertiveness behaviors are mostly related to observing sterility in the operating room, which is circulating nurses' most important duty. Thus, the circulating nurses in the current research paid more attention to assertiveness training. Lower scores in some behaviors like controlling the traffic of clinicians within the operating room and solving the problems can be due to the hierarchies within the operating rooms in Iranian hospitals [51], which was pointed out in former studies [35, 52]. Accordingly, circulating nurses do not warn surgeons in many cases, because they are at the top of the existing hierarchy. This situation can cause circulating nurses to feel uninterested in being an active team member for solving possible problems.

The present study results revealed a promotion in most task management behaviors after the intervention. The use of programmed task management (similar to the used training) can clarify standards and procedures in the operating room and improve work efficiency among circulating nurses [53]. Circulating nurses' ability to cope with stressful situations is also very important, because there are many sources of stress in the operating room [54]. The high workload in the perioperative stage can be managed by prioritizing tasks and using proper planning, which was a part of the present interventional program and resulted in improved coping skills. However, the circulating nurses did not do well enough in maintaining some standards after the intervention such as not using mobile phones and avoiding unnecessary exits from the operating rooms. Other intervention types such as policymaking can be helpful in this regard.

As mentioned above, some of the behaviors were not significantly improved or had still low scores after the educational intervention. Considering some behaviors, there might be a cultural resistance against the adoption of the learned training [39]. In the current study, the researchers made genuine attempts to suggest specific aspects of the intervention that could be useful for different behaviors. This issue was recommended in a previous study, as well [1]. Yet, it seems that educational intervention may not be sufficient for improvement of all nontechnical skills, and policymaking can be considered another way for enhancing these behaviors [35]. Correcting the existing hierarchies can also be helpful and let circulating nurses show their skills. In other words, if training cannot be helpful in improving a given behavior, other managerial solutions can be used and investigated in future studies [1]. Using sufficient educational materials about nontechnical skills in the early years of training circulating nurses can also be useful. It has been proved that undergraduate education regarding nontechnical skills provides basic understandings of the factors influencing human performance [39].

In the current research, the younger and less experienced circulating nurses showed significantly higher improvements in their scores of nontechnical skills compared to those who were older and more experienced. This could be associated with younger people's higher ability to learn [55]. On the other hand, more experienced circulating nurses may tend to be overconfident and feel a lower need for learning nontechnical skills. Furthermore, the results indicated that the educational intervention was more effective among females, which could result from their higher openness to learning [56]. This result could also be attributed to the fact that females tend to have a higher motivation for learning and breaking the cycle of possible gender inequalities. Another study also concluded that learning engagement was higher in females than in males [57]. Moreover, the present study findings revealed that the educational intervention was significantly more effective among the circulating nurses with Bachelor's degrees in comparison to those with Associate degrees. Generally, educated people tend to have healthier and safer behaviors [58]. Considering the role of demographics in the effectiveness of educational interventions, other changes such as policymaking and supervision can be used as the complement.

This study had some limitations. Firstly, the retention of the learned nontechnical skills can degrade over time. Hence, analyzing the outcomes of the trained behaviors can be a more useful way to assess the improvements. Secondly, the circulating nurses might have acted differently due to the presence of the observer.

## 5. Conclusion

Circulating nurses need to use nontechnical skills to perform safely and efficiently. Educational intervention is a useful way to improve circulating nurses' nontechnical skills. However, improving some of these skills needs other changes such as setting policies, correcting the existing hierarchies in the operating room, retraining programs, and using sufficient educational materials about nontechnical skills in the early years of training circulating nurses.

## Figures and Tables

**Table 1:** Circulating nurse's nontechnical skills framework.

Nontechnical skill domain	Elements
Task management	Maintaining operating room standards
	Coping with stress
	Proper planning and preparing the setting
Teamwork	Cooperation
	Coordination
Situational awareness	Checking the necessary elements
	Seeking the process of surgery
	Anticipation
Communication	Exchange information
	Communication management
Leadership	Assertiveness
	Controlling the operating room situation

**Table 2:** The description of the rating scale for the CPLINTS items.

Score	Label	Description
0	Necessary but not observed	The behavior is necessary but does not take place at all.
1	Poor	Performance is not acceptable and can potentially endanger patient safety; remedial action is required.
2	Marginal	Performance indicates reasons for concern; considerable improvement is needed.
3	Acceptable	Performance is of a satisfactory standard but can be improved.
4	Excellent	Performance is of a high standard level and enhances patient safety.
Not applicable	NA	The behavior is not needed during surgery.

**Table 3:** The demographics of the study population.

Variable		No intervention group	Intervention group
Age (years)^∗^		31 (10)	33 (11)
Work experience (years)^∗^		8 (12)	10 (12)
Gender^∗∗^	Male	70 (46.66%)	67 (44.66%)
	Female	80 (53.34%)	83 (55.34%)
Education level^∗∗^	Associate degree	40 (26.66%)	35 (23.33%)
	Bachelor's degree	98 (65.34%)	105 (70%)
	Master's degree	12 (8%)	10 (6.67%)

^∗^Median (interquartile range). ^∗∗^Frequency (percent).

**Table 4:** Comparison of the scores of different nontechnical skills within groups and postintervention scores between groups.

Domain	No intervention group Median (IQR)^∗^			Intervention group Median (IQR)^∗^			Mann–Whitney *U* test results (postintervention scores)	
	Before	After	Difference	Before	After	Difference	*Z*-score	*p* value
Task management	2.15 (0.38)	2.21 (0.45)	0.06 (0.31)	2.07 (0.54)	2.71 (0.31)	0.64 (0.53)	9.12^∗∗^	<0.001^∗∗∗^
Teamwork	2.14 (0.57)	2.14 (0.50)	0.00 (0.29)	2.00 (0.62)	2.85 (0.43)	0.84 (0.48)	8.64^∗∗^	<0.001^∗∗∗^
Situational awareness	2.57 (0.57)	2.42 (0.42)	-0.14 (0.44)	2.57 (0.29)	3.00 (0.43)	0.42 (0.29)	9.48^∗∗^	<0.001^∗∗∗^
Communication	2.25 (0.38)	2.45 (0.38)	0.20 (0.55)	2.37 (0.40)	3.00 (0.56)	0.62 (0.44)	9.21^∗∗^	<0.001^∗∗∗^
Leadership	1.71 (0.38)	1.82 (0.31)	0.11 (0.32)	1.75 (0.63)	2.12 (0.33)	0.37 (0.83)	7.05^∗∗^	<0.001^∗∗∗^
Overall score	2.16 (0.18)	2.26 (0.22)	0.09 (0.41)	2.14 (0.29)	2.73 (0.33)	0.59 (0.40)	12.04^∗∗^	<0.001^∗∗∗^

^∗^Interquartile range; ^∗∗^absolute value; ^∗∗∗^significant.

**Table 5:** The improvement of the domains' scores in the intervention group.

Nontechnical skill domains	Improvement of scores
Task management	30.9%
Teamwork	42.5%
Situational awareness	16.7%
Communication	26.6%
Leadership	21%
Overall score	27.5%

## Data Availability

Data will be available on reasonable requests.

## References

[1] Gordon M., Darbyshire D., Baker P. (2012). Non-technical skills training to enhance patient safety: a systematic review. *Medical Education*.

[2] Rostamabadi A., Zamanian Z., Sedaghat Z. (2017). Factors associated with work ability index (WAI) among intensive care units’ (ICUs’) nurses. *Journal of Occupational Health and Epidemiology*.

[3] Sharma B., Mishra A., Aggarwal R., Grantcharov T. P. (2011). Non-technical skills assessment in surgery. *Surgical Oncology*.

[4] Amano M., Kitabatake T., Nakata O. (2020). Development of MRI projection mapping system for breast-conserving surgery in the operating room: preliminary clinical results in invasive breast cancer. *BioMed Research International*.

[5] Gasparini G., Torroni A., di Nardo F. (2015). OSAS surgery and postoperative discomfort: phase I surgery versus phase II surgery. *BioMed Research International*.

[6] Kim T. T., Johnson J. P., Pashman R., Drazin D. (2016). Minimally invasive spinal surgery with intraoperative image-guided navigation. *BioMed Research International*.

[7] di Matteo B., Angele P., Lattermann C., Kon E. (2018). Innovative techniques to enhance musculoskeletal surgery outcomes. *BioMed Research International*.

[8] Doumouras A., Hamidi M., Lung K. (2017). Non-technical skills of surgeons and anaesthetists in simulated operating theatre crises. *British Journal of Surgery*.

[9] Uramatsu M., Fujisawa Y., Mizuno S., Souma T., Komatsubara A., Miki T. (2017). Do failures in non-technical skills contribute to fatal medical accidents in Japan? A review of the 2010–2013 national accident reports. *BMJ Open*.

[10] Somasundram K., Spence H., Colquhoun A. J., Mcilhenny C., Biyani C. S., Jain S. (2018). Simulation in urology to train non-technical skills in ward rounds. *BJU International*.

[11] Pucher P. H., Aggarwal R., Batrick N., Jenkins M., Darzi A. (2014). Nontechnical skills performance and care processes in the management of the acute trauma patient. *Surgery*.

[12] Krage R., Zwaan L., Tjon Soei Len L. (2017). Relationship between non-technical skills and technical performance during cardiopulmonary resuscitation: does stress have an influence?. *Emergency Medicine Journal*.

[13] Cooper W. O., Spain D. A., Guillamondegui O. (2019). Association of coworker reports about unprofessional behavior by surgeons with surgical complications in their patients. *JAMA Surgery*.

[14] Kalantari R., Zamanian Z., Hasanshahi M. (2021). Development and psychometric evaluation of a behavioral marker system for circulating nurse's non-technical skills. *Perioperative Care and Operating Room Management*.

[15] Thomas M. J. (2004). Predictors of threat and error management: identification of core nontechnical skills and implications for training systems design. *The International Journal of Aviation Psychology*.

[16] Hollands M. (2013). Providing non-technical skills for surgeons. *The Medical Journal of Australia*.

[17] Hu Y.-Y., Parker S. H., Lipsitz S. R. (2016). Surgeons' leadership styles and team behavior in the operating room. *Journal of the American College of Surgeons*.

[18] Griffin C., Aydın A., Brunckhorst O. (2020). Non-technical skills: a review of training and evaluation in urology. *World Journal of Urology*.

[19] Nicolaides M., Cardillo L., Theodoulou I. (2018). Developing a novel framework for non-technical skills learning strategies for undergraduates: a systematic review. *Annals of medicine and surgery*.

[20] Hull L., Arora S., Aggarwal R., Darzi A., Vincent C., Sevdalis N. (2012). The impact of nontechnical skills on technical performance in surgery: a systematic review. *Journal of the American College of Surgons*.

[21] WHO (2009). *WHO guidelines for safe surgery: safe surgery saves lives*.

[22] Redaelli I. (2018). Nontechnical skills of the operating theatre circulating nurse: an ethnographic study. *Journal of Advanced Nursing*.

[23] Kalantari R., Zamanian Z., Jamali J., Faghihi A., Hasanshahi M., Gheysari S. (2021). Reviewing the existing observational tools for assessment of circulating nurses' nontechnical skills. *Journal of Pediatric Surgical Nursing*.

[24] Yang Y. T., Henry L., Dellinger M., Yonish K., Emerson B., Seifert P. C. (2012). The circulating nurse's role in error recovery in the cardiovascular OR. *AORN Journal*.

[25] Ribakova A., Deklava L. (2018). Assessment of non-technical skills of operating room nurses. *SHS Web of Conferences*.

[26] Garosi E., Kalantari R., Zanjirani Farahani A., Zuaktafi M., Hosseinzadeh Roknabadi E., Bakhshi E. (2020). Concerns about verbal communication in the operating room: a field study. *Human Factors*.

[27] Riley R. G., Manias E. (2006). Governance in operating room nursing: nurses' knowledge of individual surgeons. *Social Science & Medicine*.

[28] Epstein R. M., Hundert E. M. (2002). Defining and assessing professional competence. *JAMA*.

[29] McCulloch P., Mishra A., Handa A., Dale T., Hirst G., Catchpole K. (2009). The effects of aviation-style non-technical skills training on technical performance and outcome in the operating theatre. *Quality & Safety in Health Care*.

[30] Matharoo M., Haycock A., Sevdalis N., Thomas-Gibson S. (2014). Endoscopic non-technical skills team training: the next step in quality assurance of endoscopy training. *World Journal of Gastroenterology*.

[31] Yule S., Parker S. H., Wilkinson J. (2015). Coaching non-technical skills improves surgical residents' performance in a simulated operating room. *Journal of Surgical Education*.

[32] Skelton T., Nshimyumuremyi I., Mukwesi C., Whynot S., Zolpys L., Livingston P. (2016). Low-cost simulation to teach anesthetists’ non-technical skills in Rwanda. *Anesthesia Analgesia*.

[33] Berwick D. (2013). *A Promise to Learn–A Commitment to Act: Improving the Safety of Patients in England*.

[34] Kalantari R., Farahani A. Z., Garosi E., Badeli H., Jamali J. (2019). Translation and psychometric properties of the Persian version of Oxford non-technical skills 2 system: assessment of surgical teams' non-technical skills in orthopedic surgery wards. *Archives of Bone and Joint Surgery*.

[35] Kalantari R., Zakerian S. A., Mahmodi Majdabadi M., Zanjirani Farahani A., Meshkati M., Garosi E. (2016). Assessing the teamwork among surgical teams of hospitals affiliated to social security organizations in Tehran City. *Journal of El Hospital*.

[36] de Vries A. H., Schout B. M., van Merriënboer J. J. (2017). High educational impact of a national simulation-based urological curriculum including technical and non-technical skills. *Surgical Endoscopy*.

[37] Rashid P., Gianduzzo T. (2016). Urology technical and non-technical skills development: the emerging role of simulation. *BJU International*.

[38] Zamanian Z., Kakooei H., Ayattollah S. M. T., Dehghani M. (2010). Effect of bright light on shift work nurses in hospitals. *Pakistan Journal of Biological Sciences*.

[39] Flin R., Patey R. (2009). Improving patient safety through training in non-technical skills. *BMJ*.

[40] Haller G., Morales M., Pfister R. (2008). Improving interprofessional teamwork in obstetrics: a crew resource management based training programme. *Journal of Interprofessional Care*.

[41] Schutte P. C., Trujillo A. C. (1996). Flight crew task management in non-normal situations. *Proceedings of the Human Factors and Ergonomics Society Annual Meeting*.

[42] Yule S., Flin R., Paterson-Brown S., Maran N., Rowley D. (2006). Development of a rating system for surgeons' non-technical skills. *Medical Education*.

[43] Graafland M., Schraagen J. M., Boermeester M. A., Bemelman W. A., Schijven M. P. (2015). Training situational awareness to reduce surgical errors in the operating room. *The British Journal of Surgery*.

[44] Bleakley A., Allard J., Hobbs A. (2012). Towards culture change in the operating theatre: embedding a complex educational intervention to improve teamwork climate. *Medical Teacher*.

[45] Sonoda Y., Onozuka D., Hagihara A. (2018). Factors related to teamwork performance and stress of operating room nurses. *Journal of Nursing Management*.

[46] Takala R. S. K., Pauniaho S. L., Kotkansalo A. (2011). A pilot study of the implementation of WHOSurgical Checklist in Finland: improvements in activities and communication. *Acta Anaesthesiologica Scandinavica*.

[47] Gardezi F., Lingard L., Espin S., Whyte S., Orser B., Baker G. R. (2009). Silence, power and communication in the operating room.

[48] Amato-Vealey E. J., Barba M. P., Vealey R. J. (2008). Hand-off communication: a requisite for perioperative patient safety. *AORN Journal*.

[49] Berkenstadt H., Haviv Y., Tuval A. (2008). Improving handoff communications in critical care: utilizing simulation-based training toward process improvement in managing patient risk. *Chest*.

[50] Rydenfält C., Johansson G., Odenrick P., Åkerman K., Larsson P. A. (2015). Distributed leadership in the operating room: a naturalistic observation study. *Cognition, Technology & Work*.

[51] Hasanshahi M., Kalantari R., Zamanian Z., Gheysari S., Bakhshi E. (2020). Assessment of non-technical skills in Iranian orthopedic surgeons: an observational study. *Journal of Advances in Medical and Biomedical Research*.

[52] Lingard L., Espin S., Whyte S. (2004). Communication failures in the operating room: an observational classification of recurrent types and effects. *BMJ Quality and Safety*.

[53] Ling H., Ge W., Min H., Xiao-Yan P. (2011). Application of programmed management in the task of circulating nurse in operating room. *Journal of Nursing Science*.

[54] Smith J., Palesy D. (2018). Technology stress in perioperative nursing: an ongoing concern. *Journal of Perioperative Nursing*.

[55] Janacsek K., Fiser J., Nemeth D. (2012). The best time to acquire new skills: age-related differences in implicit sequence learning across the human lifespan. *Developmental Science*.

[56] Severiens S. E., ten Dam G. T. M. (1994). Gender differences in learning styles: a narrative review and quantitative meta-analysis. *Higher Education*.

[57] Korlat S., Kollmayer M., Holzer J. (2021). Gender differences in digital learning during COVID-19: competence beliefs, intrinsic value, learning engagement, and perceived teacher support. *Frontiers in Psychology*.

[58] Margolis R. (2013). Educational differences in healthy behavior changes and adherence among middle-aged Americans. *Journal of Health Social Behavior*.

